# Orthotropic Behavior of Twin-Roll-Cast and Hot-Rolled Magnesium ZAX210 Sheet

**DOI:** 10.3390/ma15186437

**Published:** 2022-09-16

**Authors:** Madlen Ullmann, Christoph Kaden, Kristina Kittner, Ulrich Prahl

**Affiliations:** Institute of Metal Forming, Technische Universität Bergakademie Freiberg, Bernhard-von-Cotta Straße 4, 09599 Freiberg, Germany

**Keywords:** orthotropic, anisotropy, yield loci, Hill, Lankford, localization, AZ31, magnesium, sheet

## Abstract

Magnesium sheet metal alloys offer a deformation asymmetry, which is strongly related to grain size and texture. In order to predict deformation behavior as well as to provide methods to eliminate anisotropy and yield asymmetry, a lot of effort is invested in studying the tension–compression asymmetry of magnesium alloys. However, only a few studies deal with the characterization of the yield asymmetry of magnesium wrought alloys, especially Ca-containing alloys, related to temperature and strain. In this study, the orthotropic behavior of a twin-roll-cast, homogenized, rolled and finish-annealed Mg-2Zn-1Al-0.3Ca (ZAX210) magnesium alloy was investigated by tensile testing at room temperature, 150 °C and 250 °C. The *r*-values were determined and the Hill’48 yield criterion was used for the constitutive formulation of the plastic yielding and deformation. The yield loci calculated using Mises and Hill’48 as well as the determined *r*-values reveal an almost isotropic behavior of the ZAX210 alloy. The r-value increases with increasing logarithmic strain. At 0.16 logarithmic strain the *r*-values at room temperature vary between 1 (0°) and 1.5 (45° and 90°). At higher temperatures (250 °C), *r*-values close to 1 at all tested directions are attained. The enhanced yield asymmetry can be attributed to the weaker basal texture that arises during hot rolling and final annealing of the twin-roll-cast ZAX210 magnesium alloy. In comparison to AZ31, the ZAX210 alloy shows a yield behavior close to transversal isotropy. Finally, responsible mechanisms for this behavior are discussed.

## 1. Introduction

The use of magnesium alloys has a positive impact on energy efficiency in the automotive and aerospace industries. In order to promote the industrial use of magnesium materials produced by forming technology, one focus of research in recent years has been on the development of magnesium alloys with improved formability. Sheet metal forming processes with multiaxial loading are used for manufacturing large-area products in mass production with tailored mechanical and technological property profiles. In order to describe the hardening behavior, for example as a basis for forming simulation, it is necessary to establish a suitable flow criterion. The basis for this is provided by investigations of magnesium sheets under biaxial loading to support the selection of the anisotropic yield criterion parameters [1,2].

The anisotropic nature of the hexagonal closed-packed structure of conventional magnesium alloys leads to the formation of strong basal textures during deformation or thermomechanical processing. Related studies have shown that these textures are mainly responsible for the formation of yield asymmetry, since during the deformation parallel to the c-axis is the formation of tensile twinning of the dominant deformation mechanism [3,4,5,6]. Davis et al. (2019) [3] showed the most effective strategies to reduce yield asymmetry and anisotropy by texture weakening and solid solution strengthening in Mg-Y alloys. Comparable effects were demonstrated by Kamrani and Fleck (2014) [5], showing that a pronounced weakening of the texture in ZEK100 alloyed by Ca goes along with a reduced yield asymmetry. Dobron et al. (2018) [7] describe that the formation of extension twins during extrusion along the extrusion direction leads to a significant reduction in the compressive yield strength, while the tensile yield strength is not influenced. The authors reveal that this behavior is related to twinning activity in the ZX10 magnesium alloy. The combination of pre-compression and subsequent heat treatment additionally causes the formation of a yield plateau. According to [7], this is due to the propagation of twins beyond grain boundaries, which is associated with the stress relaxation mechanism after strengthening of the material by the presence of a relatively high dislocation density and fine Mg_2_Ca precipitates. A comparable behavior was presented by [8] by investigating yielding by twinning during compression of an extruded AZ31 magnesium alloy.

Calcium as an alloying element is known to weaken basal texture in magnesium alloys [5,9,10], which may result in a reduced yield asymmetry. Nakata et al. (2018) [11] presented a Mg-1.1Al-0.24Ca-1.0Mn magnesium alloy with improved compressive yield stress and a small yield asymmetry after extrusion and aging treatment. The increase in the strength was attributed to the grain size refinement and the uniform distribution of the Guinier Preston zones by aging. The addition of Mn supports the grain refinement by suppressing significant grain growth because of nano-scaled Mn-containing precipitates. Investigations of Song et al. (2020) [12] revealed that torsion deformation and aging treatment of an extruded AZ91 rod can enhance the yield strength effectively, while the yield asymmetry is eliminated. In summary, texture weakening [6,13,14], solid solution strengthening, grain size reduction [15] and precipitation strengthening [16,17] are effective strategies to develop magnesium alloys with reduced anisotropy and yield asymmetry. However, few studies address the yield asymmetry of Ca-containing magnesium alloy sheets; in particular, properties are often presented only at room temperature, not at elevated temperatures with coincident strain dependence.

The Mg-2Zn-1Al-0.3Ca (ZAX210) magnesium alloy was designed driven by the effort to develop an alloy with higher formability without using expensive Rare Earth elements. The hot rolling process leads to the formation of a fine-grained microstructure, weaker basal texture and a lower anisotropy of the mechanical properties [18]. As mentioned above, studies about the behavior of yield anisotropy are currently not available. As a result of the increasing importance of the alloy for industrial applications, the orientation dependent flow behavior of the ZAX210 magnesium alloy was investigated in order to provide basic yield model data for the numerical simulation.

## 2. Materials and Methods

### 2.1. Materials

Twin-roll-cast, rolled and finish-annealed magnesium sheets of Mg-2Zn-1Al-0.3Ca (ZAX210) alloy with a thickness of 1.5 mm were used for these investigations. The chemical composition is shown in the table below (Table 1).

The sheets were produced as described in [19]. Microstructural and texture characterization were performed using ZEISS GeminiSEM 450 device at the Institute of Metal Forming, Freiberg, Germany. The accelerating voltage for electron backscatter diffraction (EBSD) analysis was between 15 kV and 20 kV. A step size of 0.5 µm was selected. The AZtec software was used to analyze the recorded data.

### 2.2. Experimental Setup

**Figure 1:**
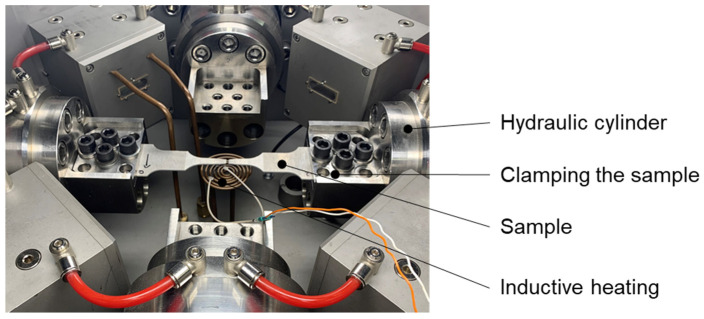
Biaxial testing device BTA-840 (BÄHR Thermoanalyse GmbH) at the Institute of Metal Forming, TU Bergakademie Freiberg.

The tensile tests to determine the LANKFORD parameters (*r*-values) were carried out on the biaxial testing device BTA-840 (TA Instruments former BÄHR Thermoanalyse GmbH, Hüllhorst, Germany) at the Institute of Metal Forming, TU Bergakademie Freiberg (Figure 1). The system is equipped with a total of four hydraulic cylinders, which are aligned at right angles in a cross shape on one plane and can be controlled individually. For the uniaxial tensile test only two cylinders are required. Induction coils are installed above and below the center of the specimen to heat the specimens. This allows a very precise specification of temperature–time curves.

**Figure 2:**
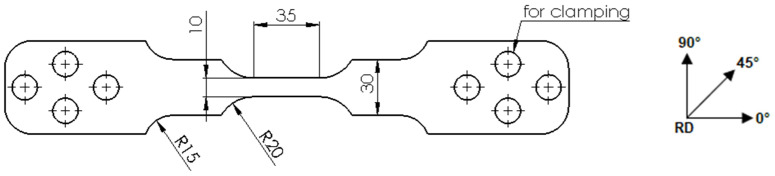
Sample dimensions and sampling directions for the uniaxial tensile tests (dimensions in mm).

To determine the *r*-values, tensile specimens were produced in the rolling direction ($r_{0{}^{\circ}}$), transverse to the rolling direction ($r_{90{}^{\circ}}$) and at an angle of 45° to the rolling direction ($r_{45{}^{\circ}}$) according to the specifications of DIN EN ISO 6892 (Figure 2). The tests were carried out at room temperature, 150 °C and 250 °C, the feed rate of the hydraulic cylinders was 0.35 mm/s, and the heating rate was 2 K/s. For a better statistical validation, three samples for each temperature and direction were tested. For the subsequent evaluation, cameras were installed above the specimens to film the tensile test. A grid was applied to the surface of the specimen to improve the detection of length and width strain.

### 2.3. Graphical Evaluation

**Figure 3:**
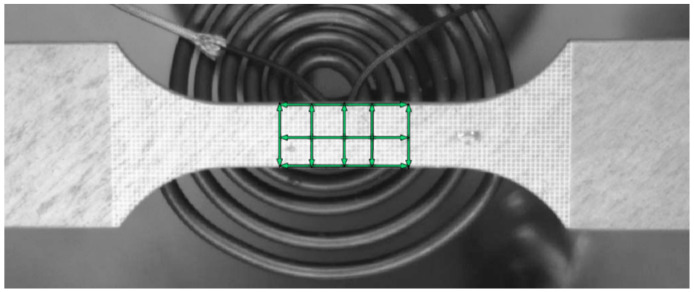
Virtual extensometer over measuring range (20 × 10 mm), GOM Correlate.

Digital image correlation software, ZEISS GOM Correlate Pro, was used to determine the deformation and calculate the *r*-values. To use the software, the filmed videos of the individual tensile tests were broken down into individual frames and were uploaded into the program. Next, virtual extensometers were placed on the specimen within the measurement range (Figure 3). A total of three marks were used for length and five for width. The program then measured the deformation of the individual images, the data were uploaded into a calculation program and the *r*-values were calculated and output as a function of the local logarithmic. Strain was calculated automatically.

### 2.4. Hill’48 Yield Criterion

Hill [20] proposed a constitutive formulation for the plastic yielding and deformation of anisotropic metals. The quadratic yield criterion is given by Equation (1):

(1) $$2f\left(\sigma \right)=F{\left(\sigma_{yy}-\sigma_{zz}\right)}^{2}+G{\left(\sigma_{zz}-\sigma_{xx}\right)}^{2}+H{\left(\sigma_{xx}-\sigma_{yy}\right)}^{2}+2\left(L\tau_{yz}^{2}+M\tau_{zx}^{2}+N\tau_{xy}^{2}\right)=1$$

where *f* is the yield function; *σ_xx_*, *σ_yy_* and *σ_zz_* are the stresses in the rolling, transverse and thickness directions; *τ_xy_*, *τ_yz_* and *τ_zx_*, are the shear stresses in the *xy*, *yz* and *zx* planes, respectively. The Hill’48 material parameters *F*, *G*, *H*, *L*, *M* and *N* are the constants that define the anisotropy of the material. When applying this yield criterion to sheet metal, it can be simplified under the assumption of plane stress condition (*σ_xx_ = τ_yz_ = τ_zx_* = 0):

(2) $$2f\left(\sigma \right)=\left(G+H\right)\sigma_{xx}^{2}+\left(F+H\right)\sigma_{yy}^{2}-2H\sigma_{xx}\sigma_{yy}+2N\tau_{xy}^{2}.$$

In this study, the identification of the anisotropy parameters for the Hill’48 model was carried out by applying the classical approach, which uses the *r*-values from three uniaxial tension tests (0°, 45° and 90° to the rolling direction) at different temperatures:

(3) $$F=\frac{r_{0}}{r_{90}\left(1+r_{0}\right)}$$

(4) $$G=\frac{1}{2}\left(1+\frac{1-r_{0}}{1+r_{0}}\right)$$

(5) $$H=\frac{1}{2}\left(1+\frac{r_{0}-1}{1+r_{0}}\right)$$

(6) $$L=M=\frac{3}{2}$$

(7) $$N=\frac{3}{2}\left(\frac{\left(1+2r_{45}\right)\left(r_{0}+r_{90}\right)}{3r_{90}\left(1+r_{0}\right)}\right)$$

In the present work, the tension–compression asymmetry method is not used because:

(1) When the sheet metal is deep drawn, the biaxial compressive stress in the sheet metal plane will not cause the so-called “thickening” of the sheet metal. If there is surface compressive stress, only wrinkles are formed, which macroscopically combine to form a larger sheet cross-section due to the high pressure in the press.
(2) In the special case of the inner bending radius, the small appearance of compressive stress can be neglected in the modeling.
(3) The manufacturing tolerance of a cylindrical compression specimen in the plane of the plate (direction of compression equal to direction of sheet thickness) is too large compared to the plate with a thickness of 1.5 mm, and the calculation of the flow curve will be accompanied by a large error.

## 3. Results and Discussion

### 3.1. Microstructure and Texture of the Initial State

**Figure 4:**
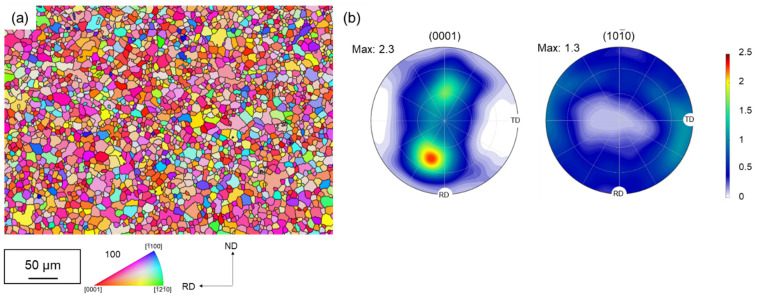
Microstructure and texture of the ZAX210 sheets: (**a**) EBSD-Map and (**b**) pole figures.

Twin-roll-cast, rolled and finish-annealed magnesium sheets of Mg-2Zn-1Al-0.3Ca (ZAX210) alloy with a thickness of 1.5 mm were used for the investigations in this study. Figure 4 presents the EBSD map and the texture of the ZAX210 sheets. After final annealing, the sheets offer a microstructure with recrystallized equiaxed grains exhibiting an average grain size of 8.3 µm (±4.0 µm). The hot rolling and final annealing lead to the formation of a basal texture. The texture reveals for Ca-containing magnesium alloys a characteristic basal pole split in the rolling direction (RD) as well as a broadening of the texture components in the RD and transverse direction (TD), the latter being less pronounced. In general, the texture components have a low intensity. The c-axis of the hexagonal cells is tilted by 35° to 45° from the normal direction (ND) to RD. The prismatic pole intensity is aligned in the transverse direction without showing a six-fold symmetry of the maximum pole intensities across ND. This can be attributed to the pronounced basal pole broadening in RD.

### 3.2. Variation in r-Values with Logarithmic Strain and Temperature and Hill’48 Coefficients

For the calculation of the *r*-values, the logarithmic strain up to the necking was used, whereby this range becomes smaller with increasing temperature. The *r*-values as a function of the local logarithmic strain are shown in Figure 4. The *r*-values are small (0.5 to 0.7) during the early stage of deformation and increase with local logarithmic strain. At all tested temperatures, there is a tendency that *r*_90°_ > *r*_45°_ > *r*_0°_. However, the difference between *r*_90°_ and r_45°_ is very small (maximum deviation is 10%). At room temperature, the *r*-value at a logarithmic strain of 0.16 is 1 (0°) and 1.5 (45°, 90°), respectively. With increasing the temperature to 150 °C the *r*-value also rises to 1.2 (0°) and 1.5 to 1.65 (45°, 90°). The *r*-values at 250 °C are generally lower and reach values close to 1. The obtained *r*-values are a direct result of the crystallographic texture present in the ZAX210 alloy, combined with the relative resolved shear strengths of the slip and twinning systems.

The plane anisotropy Δ*r* is between −0.2 and 0 at all temperatures. These values indicate a slight anisotropic behavior in the sheet plane and lead to earing formation at an angle of 45° to the rolling direction. The maximum values for the mean vertical anisotropy *r_m_* are 1.4 for RT, 1.5 for 150 °C and 1.1 for 250 °C. This gradual increase in *r*-value from the RD (rolling direction, 0°) to the TD (transverse direction, 90° to RD) is consistent with results for the AZ31 alloy from the literature, e.g., [21]. Yi et al. (2010) [22] explain the strength dependence of the *r*-value in AZ31 with the tilting of the basal planes. The more they are tilted in the direction of deformation, the more likely the activation of <a>-dislocations of the basal system can occur and strain in the c-axis direction can be realized, since the *r*-value is inversely proportional to the strain in the thickness direction. The variation in the *r*-values (0°, 45° and 90°) of the ZAX210 sheets can also be explained by their texture (see texture of ZAX210 sheets in [18] and Figure 4). Loading in the TD is advantageous for basal <a>-slip, since the tilted basal planes and the tilt angle is higher in the TD than in the RD. Therefore, the yield strength of TD is lower than that of RD. Compared with the *r*-value published for AZ31, the *r*-value close to 1 in alloy ZAX210 indicates that the cross-sectional contraction occurs isotropically, i.e., the strains in the width and thickness directions are similar. The achievement of *r*-values close to 1 is due to the activation of non-basal slip systems. Already, Duygulu et al. (2003) [23] showed in their simulations that only the activation of additional non-basal slip systems, especially the <c + a> pyramidal planes, leads to an increasing uniformity of the deformation, so that the *r*-value drops to the value close to isotropy (*r*~1).

**Figure 5:**
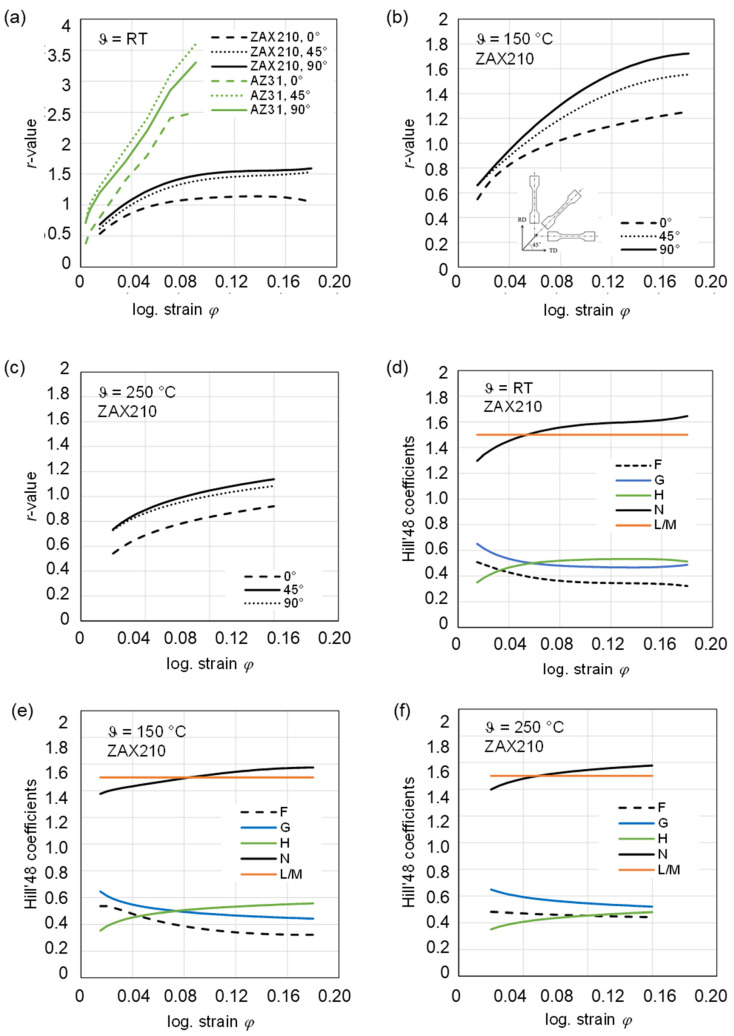
Variation in *r*-values with log. strain used to determine the material parameters for the anisotropic yield functions and Hill’48 coefficients: (**a**) ZAX210 in comparison with AZ31 (data from [24]) at RT, (**b**) ZAX210 at 150 °C, (**c**) ZAX210 at 250 °C, (**d**) Hill’48 coefficients for ZAX210 at RT, (**e**) Hill’48 coefficients for ZAX210 at 150 °C and (**f**) Hill’48 coefficients for ZAX210 at 250 °C.

With help of the *r*-values, the coefficients for the Hill’48 criteria could be determined. The graphs of the Hill’48 coefficients as a function of logarithmic strain are comparable for all temperatures and are shown for room temperature, 150 °C and 250 °C for the ZAX210 alloy in Figure 5. The specific values of the Hill’48 coefficients at room temperature are shown in Table 2.

### 3.3. Yield Loci Calculated Using Mises and Hill’48

**Figure 6:**
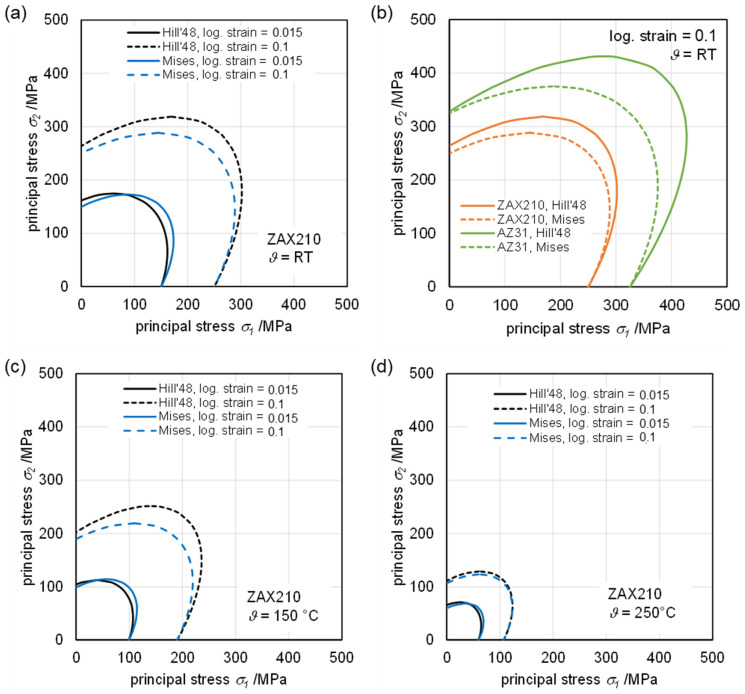
Yield loci of ZAX210 calculated with Hill’48 considering the *r*-values at different logarithmic strains, partly in comparison with Mises: (**a**) at room temperature, (**b**) at room temperature in comparison with AZ31, (**c**) at 150 °C, (**d**) at 250 °C.

Figure 6 shows the calculated yield loci using von Mises and Hill’48 yield functions depending on logarithmic strain and temperature for the ZAX210 sheets. As the temperature increases, the *r*-values approach 1, resulting in isotropic behavior and thus Hill’48 and Mises converge. Likewise, the influence of logarithmic strain onto the *r*-value decreases with increasing temperature and almost disappears at 250 °C. Compared to AZ31 [24], ZAX210 shows a significantly more isotropic behavior. In the case of ZAX210, an approximately planar isotropic material behavior at a low temperature of 150 °C can be assumed even for moderately higher strains compared to AZ31.

Ultimately, it can be stated that the in-plane material flow behavior can be identified as orthotropic with decreasing anisotropy when forming temperature is rising. In comparison to AZ31, the ZAX210 alloy shows a yield behavior close to transversal isotropy, in particular for higher temperatures, when additional shear planes might be assumed to be activated.

## 4. Conclusions

In this study the orientation dependent flow behavior of the ZAX210 magnesium alloy was investigated in order to provide basic yield model data for the numerical simulation. The following conclusions were obtained.

(1) The *r*-values are small (0.5 to 0.7) during the early stage of deformation and increase with local logarithmic strain. At all tested temperatures, there is a tendency that *r*_90°_ > *r*_45°_ > *r*_0°_.
(2) The obtained *r*-values are a direct result of the crystallographic texture present in the ZAX210 alloy, combined with the relative resolved shear strengths of the slip and twinning systems.
(3) The plane anisotropy Δ*r* is between 0 and–0.2 at all tested temperatures. These values indicate a slight anisotropic behavior in the sheet plane.
(4) The calculated yield loci using von Mises and Hill’48 yield functions depending on logarithmic strain and temperature for the ZAX210 sheets reveal an isotropic behavior, where Hill’48 and Mises converge and an approximately planar isotropic material behavior at low temperature 150 °C can be assumed even for moderately higher strains compared to AZ31.
(5) The in-plane material flow behavior can be identified as orthotropic with decreasing anisotropy at elevated temperatures.

## Figures and Tables

**Table 1:** Chemical composition of the ZAX210 sheets (wt.%) measured by optical emission spectrometry [19].

Zn	Al	Ca	Mn	Cu	Fe	Ni	Others	Mg
2.290	0.920	<0.250	0.040	0.001	0.005	0.001	<0.045	Bal.

**Table 2:** Anisotropic parameters in the Hill’48 yield function for rolled ZAX210 alloys at room temperature.

Log. Strain	F	G	H	L	M	N
0.015	0.5079	0.6511	0.3489	1.5	1.5	1.2977
0.05	0.4043	0.5123	0.877	1.5	1.5	1.4878
0.1	0.3499	0.4724	0.5276	1.5	1.5	1.5791
0.18	0.3222	0.4872	0.5128	1.5	1.5	1.6454

## Data Availability

Not applicable.

## References

[1] Hama T., Takuda H. (2012). Crystal plasticity finite-element simulation of work-hardening behavior in a magnesium alloy sheet under biaxial tension. Comput. Mater. Sci..

[2] Shi B., Yang C., Peng Y., Zhang F., Pan F. (2022). Anisotropy of wrought magnesium alloys: A focused overview. J. Magnes. Alloy..

[3] Davis A.E., Robson J.D., Turski M. (2019). Reducing yield asymmetry and anisotropy in wrought magnesium alloys—A comparative study. Mater. Sci. Eng. A.

[4] Drozdenko D., Bohlen J., Yi S.B., Minárik P., Chmelík F., Dobroň P. (2016). Investigating a twinning–detwinning process in wrought Mg alloys by the acoustic emission technique. Acta Mater..

[5] Kamrani S., Fleck C. (2014). Effects of calcium and rare-earth elements on the microstructure and tension–compression yield asymmetry of ZEK100 alloy. Mater. Sci. Eng. A.

[6] Wang J., Li X., Jin P., Li S., Ma G., Zhao L. (2018). Reducing the tension-compression yield asymmetry in an extruded ZK60 alloy by ultrafine grains. Mater. Res. Express.

[7] Dobroň P., Drozdenko D., Olejňák J., Hegedüs M., Horváth K., Veselý J., Bohlen J., Letzig D. (2018). Compressive yield stress improvement using thermomechanical treatment of extruded Mg-Zn-Ca alloy. Mater. Sci. Eng. A.

[8] Barnett M.R., Nave M.D., Ghaderi A. (2012). Yield point elongation due to twinning in a magnesium alloy. Acta Mater..

[9] Langelier B., Nasiri A.M., Lee S.Y., Gharghouri M.A., Esmaeili S. (2015). Improving microstructure and ductility in the Mg–Zn alloy system by combinational Ce–Ca microalloying. Mater. Sci. Eng. A.

[10] Stanford N. (2010). The effect of calcium on the texture, microstructure and mechanical properties of extruded Mg–Mn–Ca alloys. Mater. Sci. Eng. A.

[11] Nakata T., Xu C., Ajima R., Matsumoto Y., Shimizu K., Sasaki T.T., Hono K., Kamado S. (2018). Improving mechanical properties and yield asymmetry in high-speed extrudable Mg-1.1Al-0.24Ca (wt%) alloy by high Mn addition. Mater. Sci. Eng. A.

[12] Song B., Wang C., Guo N., Pan H., Xin R. (2017). Improving Tensile and Compressive Properties of an Extruded AZ91 Rod by the Combined Use of Torsion Deformation and Aging Treatment. Materials.

[13] Agnew S.R., Brown D.W., Tome C.N. (2006). Validating a polycrystal model for the elastoplastic response of magnesium alloy AZ31 using in situ neutron diffraction. Acta Mater..

[14] Homma T., Mendis C.L., Hono K., Kamado S. (2010). Effect of Zr addition on the mechanical properties of as-extruded Mg–Zn–Ca–Zr alloys. Mater. Sci. Eng. A.

[15] Robson J.D., Twier A.M., Lorimer G.W., Rogers P. (2011). Effect of extrusion conditions on microstructure, texture, and yield asymmetry in Mg–6Y–7Gd–0.5wt%Zr alloy. Mater. Sci. Eng. A.

[16] Robson J.D., Stanford N., Barnett M.R. (2011). Effect of precipitate shape on slip and twinning in magnesium alloys. Acta Mater..

[17] Sasaki T.T., Elsayed F.R., Nakata T., Ohkubo T., Kamado S., Hono K. (2015). Strong and ductile heat-treatable Mg–Sn–Zn–Al wrought alloys. Acta Mater..

[18] Ullmann M., Kittner K., Henseler T., Stöcker A., Prahl U., Kawalla R. (2019). Development of new alloy systems and innovative processing technologies for the production of magnesium flat products with excellent property profile. Procedia Manuf..

[19] Kittner K., Ullmann M., Henseler T., Kawalla R., Prahl U. (2019). Microstructure and Hot Deformation Behavior of Twin Roll Cast Mg-2Zn-1Al-0.3Ca Alloy. Materials.

[20] Hill R. (1948). A theory of the yielding and plastic flow of anisotropic metals. Proc. R. Soc. Lond. A.

[21] Agnew S.R. (2002). Plastic anisotropy of magnesium alloy AZ31B sheet. Essential Readings in Magnesium Technology.

[22] Yi S., Bohlen J., Heinemann F., Letzig D. (2010). Mechanical anisotropy and deep drawing behaviour of AZ31 and ZE10 magnesium alloy sheets. Acta Mater..

[23] Duygulu Ö., Agnew S.R. (2003). The effect of temperature and strain rate on the tensile properties of textured magnesium alloy AZ31B sheet. Magnes. Technol..

[24] Andar M.O., Kuwabara T., Steglich D. (2012). Material modeling of AZ31 Mg sheet considering variation of r-values and asymmetry of the yield locus. Mater. Sci. Eng. A.

